# Supplementary material for: Harnessing microcomb-based parallel chaos for random number generation and optical decision making
Source: Nat Commun. 2023 Jul 31;14:4590. doi: 10.1038/s41467-023-40152-w (PMC10390475; doi:10.1038/s41467-023-40152-w)
Supplement: Supplementary file 1 — Supplementary Information [file 41467_2023_40152_MOESM1_ESM.pdf]

Supplementary Information for

Harnessing microcomb-based parallel chaos for random number generation and optical decision making

Bitao Shen[1,†], Haowen Shu[1,†,*], Weiqiang Xie[2], Ruixuan Chen[1], Zhi Liu[5,6], Zhangfeng Ge[7], Xuguang Zhang[1], Yimeng Wang[1], Yunhao Zhang[1], Buwen Cheng[5,6], Shaohua Yu[1,4], Lin Chang[1,3,*], and Xingjun Wang[1,3,4,7,*]

[1]State Key Laboratory of Advanced Optical Communications System and Networks, School of Electronics, Peking University, Beijing, 100871, China.

[2]Department of Electronic Engineering, Shanghai Jiao Tong University, Shanghai, 200240, China.

[3]Frontiers Science Center for Nano-optoelectronics, Peking University, Beijing 100871, China.

[4]Peng Cheng Laboratory, Shenzhen 518055, China.

[5]State Key Laboratory on Integrated Optoelectronics, Institute of Semiconductors, Chinese Academy of Sciences, Beijing 100083, China.

[6]Center of Materials Science and Optoelectronics Engineering, University of Chinese Academy of Sciences, Beijing 100049, China.

[7]Peking University Yangtze Delta Institute of Optoelectronics, Nantong 226010, China.

[†]These authors contributed equally to this work

Corresponding authors: *haowenshu@pku.edu.cn, *linchang@pku.edu.cn, *xjwang@pku.edu.cn.

Supplementary note I Characterizing the chaotic state

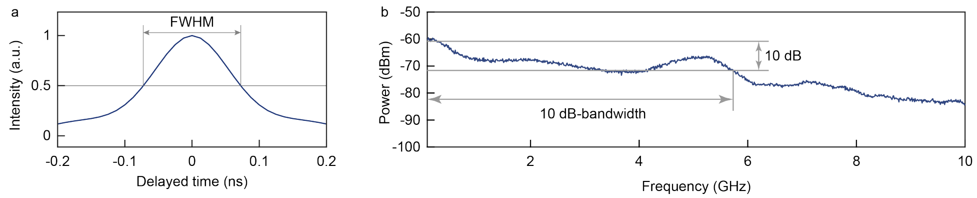

Supplementary Fig. 1: Illustration of the full-width-half-max of the autocorrelation function (**a**), and the effective radio-frequency bandwidth (**b**).

In the main part, the autocorrelation and the cross-correlation function are employed to characterize the chaotic state. Before the analysis, a detailed description of the parameter calculation is presented here. As for the time domain characterization, the auto-correlation is applied to the recorded data nevertheless in the experiment or in simulation. Considering one signal $I(t)$ captured in simulation or in experiment, the auto-correlation $ACF(\tau)$ is calculated by

$$ACF(\tau) = \frac{\langle \delta I(t+\tau) \cdot \delta I(t) \rangle_t}{\langle \delta I^2(t) \rangle_t} \tag{1}$$

Where $\delta I(t) = I(t) - \langle I(t) \rangle_t$. For ease of presentation, the full width at half maximum (FWHM) of the ACF is employed to qualify the recorded data, as shown in Supplementary Fig. 1a. A lower FWHM indicates a faster chaotic process, benefiting applications such as random bits generation and ranging.

Despite the ACF, the radio-frequency (RF) spectrum is another common characterization for the chaotic signal. As shown in Supplementary Fig. 1b, the chaotic signal obtained by detecting a comb line of the chaotic combs shows a continuous spectrum in the RF domain, with a decreasing power density apart from the zero frequency. Thus, a 10 dB-bandwidth is employed here to qualify the chaotic signal in the RF domain.

Employing the FWHM of the ACF and the 10 dB-bandwidth of the RF spectrum, we could qualify a single chaotic signal. The bandwidth is one of the most important characteristics of chaotic signal. For the detection of chaotic signals, the sampling rate should be larger than the twice of the signal bandwidth. While for applications such as random bit generation, the sampling rate can be larger than the twice of the signal bandwidth, with post-processing to obscure inter-sampling correlation [1]. While this will not increase the

rate of entropy production, which limits the random bit generation rate. The entropy rate can be estimated by:

$$h_0 = \min\left(\tau^{-1}, 2BW\right)\left(N_\epsilon - D_{KL}(p(x)\|u(x))\right) \tag{2}$$

Where τ is the sampling period, BW is the bandwidth of the entropy source, N_ϵ is the number of bits per sample, $p(x)$ is the probability density function of the entropy source, $u(x)$ is the uniform distribution and D_{KL} is the Kullback-Leibler divergence from $u(x)$ to $p(x)$. We can see that the entropy is limited by the twice of bandwidth and the probability density function. The entropy can be increased with a larger bandwidth and a more flat probability density function.

In this work, we are aimed at the realization of a massively parallel chaotic signal generator. The orthogonality between channels is of great interest to prove the parallelism. Considering two signals detected $I_m(t)$ and $I_n(t)$, the orthogonality is qualified by the cross-correlation

$$XCF_{m,n}(\tau) = \frac{\langle \delta I_m(t+\tau)\cdot\delta I_n(t)\rangle_t}{\sqrt{\langle \delta I_m^2(t)\rangle_t \cdot \langle \delta I_n^2(t)\rangle_t}} \tag{3}$$

Where $\delta I_m(t) = I_m(t) - \langle I_m(t)\rangle_t$ and $\delta I_n(t) = I_n(t) - \langle I_n(t)\rangle_t$. If I_m and I_n are correlated at a delayed time τ, there will be a peak at $XCF_{m,n}(\tau)$ where the height values the strength of the correlation. Thus, the maximum of $XCF_{m,n}(\tau)$ could be employed to value the correlation or orthogonality between two detected signals. For the ease of the expression, the maximum of $XCF_{m,n}(\tau)$ is symbolized by $XCF_{m,n}$. A lower $XCF_{m,n}$ indicates a lower correlation or a better orthogonality between $I_m(t)$ and $I_n(t)$. In the experiment, the comb lines are recorded individually by an oscilloscope to obtain $I_m(t)$ and a electrical spectrum analyzer to obtain $\tilde{I}_m(f)$). In the simulation, the comb evolution is simulated by the Lugiato-Lefever equation [2]

$$t_R \frac{\partial \mathrm{E}(t,\tau)}{\partial \mathrm{t}} = \left[-\left(\frac{\alpha}{2} - i\delta_0\right) + iL\frac{\beta_2}{2}\frac{\partial^2}{\partial\tau^2}\right]E + iL\gamma|E|^2E + \sqrt{\theta}E_{\text{in}} \tag{4}$$

with the same parameter definition shown in Methods. Based on the Lugiato-Lefever equation, we could obtain the time-domain evolution of hundreds of comb lines during a certain period.

Supplementary note II Simulation: Route into the chaotic state

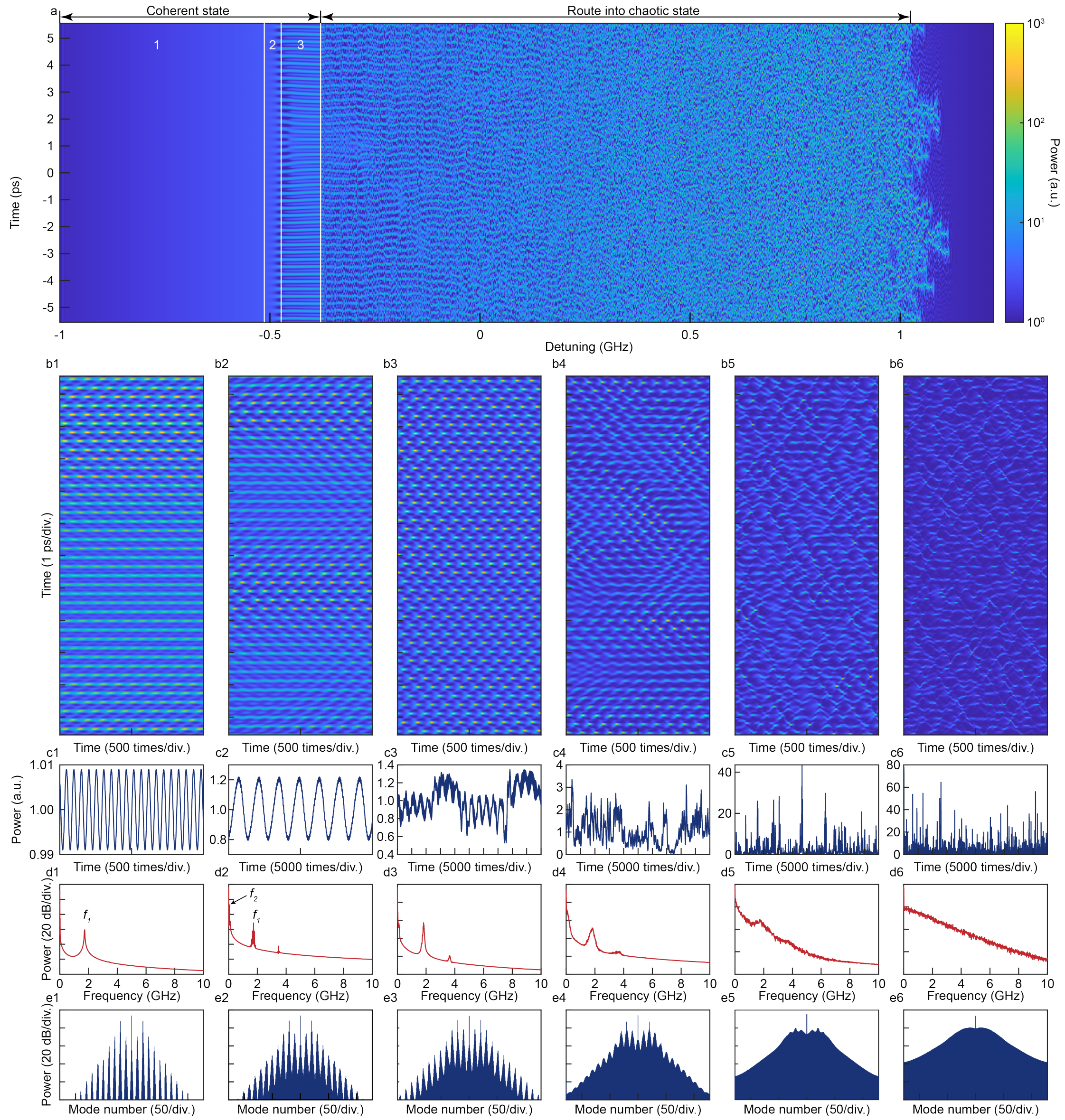

Supplementary Fig. 2: Numerical simulation of chaotic comb generation in a microcavity. **a**, the evolution of the intracavity field distribution. **b**, the intracavity field distribution evolution under different states. **c**, the time domain variation of a single comb line. **d**,the radio-frequency spectra of a single comb line. **e**,the optical spectra of different comb states.

Here, we give a universal view into the evolution of the chaotic comb in simulation. Supplementary Fig. 2a shows the intracavity field spatial distribution evolution as the pump laser tuning from higher frequency to lower frequency. Several comb states can be distinguished.

In region 1, the intracavity field is expressed as the continuous wave state or the homogeneous state. In region 2, the primary comb is stimulated, expressed as stable period multi-pulses in spatial domain. In region 3, some small comb lines, or subcomb lines, are stimulated around the primary comb lines due to the nondegenerate four-wave-mixing pumped by the pump and primary comb lines. With these small comb lines formation, multi small pulses appear between the period pulses. For states mentioned above, all stimulated comb lines are highly coherent, with the long-term stability in time domain. As more subcomb lines stimulated, the field distribution starts varying periodically with frequency f1 in time domain. As for the stimulated comb line with the highest average power, the comb line shows a sinusoidal oscillation. In RF domain, a single peak is observed. The transition from the steady state to the stable period state in time domain is realized by the Andronov-Hopf bifurcation [3]. Deeper into the resonance, peaks appear around the fundamental frequency and f_1. The time domain variation of the single comb line can be viewed as the sum of two sinusoidal oscillations with different frequency f_1 and f_2, termed as the Torus bifurcation [3]. During the transition from the stable state to the chaotic state, more peaks are stimulated (Supplementary Fig. 2c2) and broadened (Supplementary Fig. 2c3 and c4) in the RF spectra. As for the time domain of the intracavity field, the pattern in spatial-time domain follows an extended quasiperiodic route to chaos. Finally, the intracavity field shows a complex evolution (Supplementary Fig. 2c5 and c6), without distinguishable periodicity, indicating the formation of the spatiotemporal chaos.

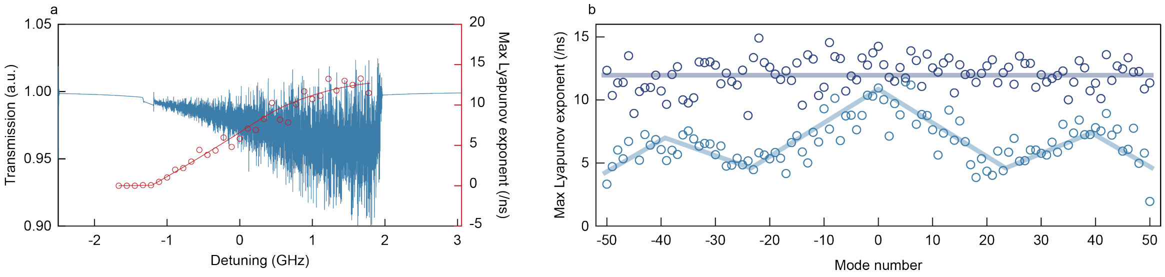

Supplementary Fig. 3: Largest lyapunov exponents of different comb states. **a**, the variation of the max lyapunov exponent with the tuning of the pump laser. **b**, the max lyapunov exponent distribution under different chaotic states.

As illustrated above, the chaotic comb would be formed as the pump laser sweeps into the resonance. This is also verified in simulation by calculating the largest lyapunov exponent

(LLE) under different comb states, as shown in Supplementary Fig. 3a. To characterize the chaos, the evolution under 10000 round-trips $I_n(t, \Delta)$ are recorded under different detuning and employed, where n indicates the mode number of comb lines, t represents the evolution time in unit of round-trip time and Δ is the detuning between the pump laser and cold cavity resonance. Under different detuning, the largest lyapunov exponent for each comb line is calculated and the max one is shown in Supplementary Fig. 3a. Deeper into the resonance, a larger LLE is obtained. Supplementary Fig. 3b shows the $LLEs$ of comb lines around the pump mode under $\Delta = 1.9$ GHz and $\Delta = 0$ GHz. Uniform chaotic properties are estimated under larger detuning. It is worth noting that a uniform power distribution is also established under the same detuning.

Supplementary note III Experiment: Route into the chaotic state

Supplementary Fig. 4a shows the 90 GHz FSR AlGaAs ring resonator tested, working in anomalous dispersion. By sweeping a continuous-wave laser through a resonance, multiple comb lines can be stimulated with different comb states, termed as the primary comb state, the subcomb state, and the chaotic comb state, as reported previously. Also, the chaotic comb state can be estimated by the destabilization of the soliton state, as discussed in [4]. To observe the path from the coherent comb state to the chaotic comb state, the evolution of the comb line around 1535 nm is recorded in time domain and radio frequency domain as the sweeping of the pump laser, as shown in Supplementary Fig. 4b. In Supplementary Fig. 4c, the regions of chaotic comb state and other states are clearly distinguishable, with the evolution of the chaotic state showing a rapid oscillation at the total output power (blue line) and the comb power (red line). Supplementary Fig. 4d shows the RF spectral variation with the pump laser tuning, where the RF spectrum evolves from multi peaks to continuous broadband. As subcombs stimulated, the comb line will express as a sinusoidal periodic oscillation in time domain, corresponding to a clear limit cycle phase portrait in Supplementary Fig. 4e1, related with the Hopf bifurcation. The sinusoidal periodic character is also verified in RF domain, which shows a remarkable single peak. With the pump laser sweeping deeper into the resonance, more secondary comb lines are stimulated, and the comb line goes into the multi-period state as shown in Supplementary Fig. 4g2, with four separated peaks. In the time domain, the variation can be well fit by the sum of two sinusoids, indicating the existence of the secondary Hopf bifurcation. With a larger detuning, more peaks appear and are broadened in the RF domain, undergoing an extended quasiperiodic route to chaos. Finally, a continuous RF spectrum is obtained as the multi peaks connecting with each other, marking the comb line evolves into a spatiotemporal chaotic state. Due to the natural complexity of the spatiotemporal chaos, it is hard to figure out the chaotic attractor in the 2D phase portrait. Thus, there is no significant periodicity in the time domain variation of comb lines. The evolution process agrees well with simulation results shown in the Supplementary Note II.

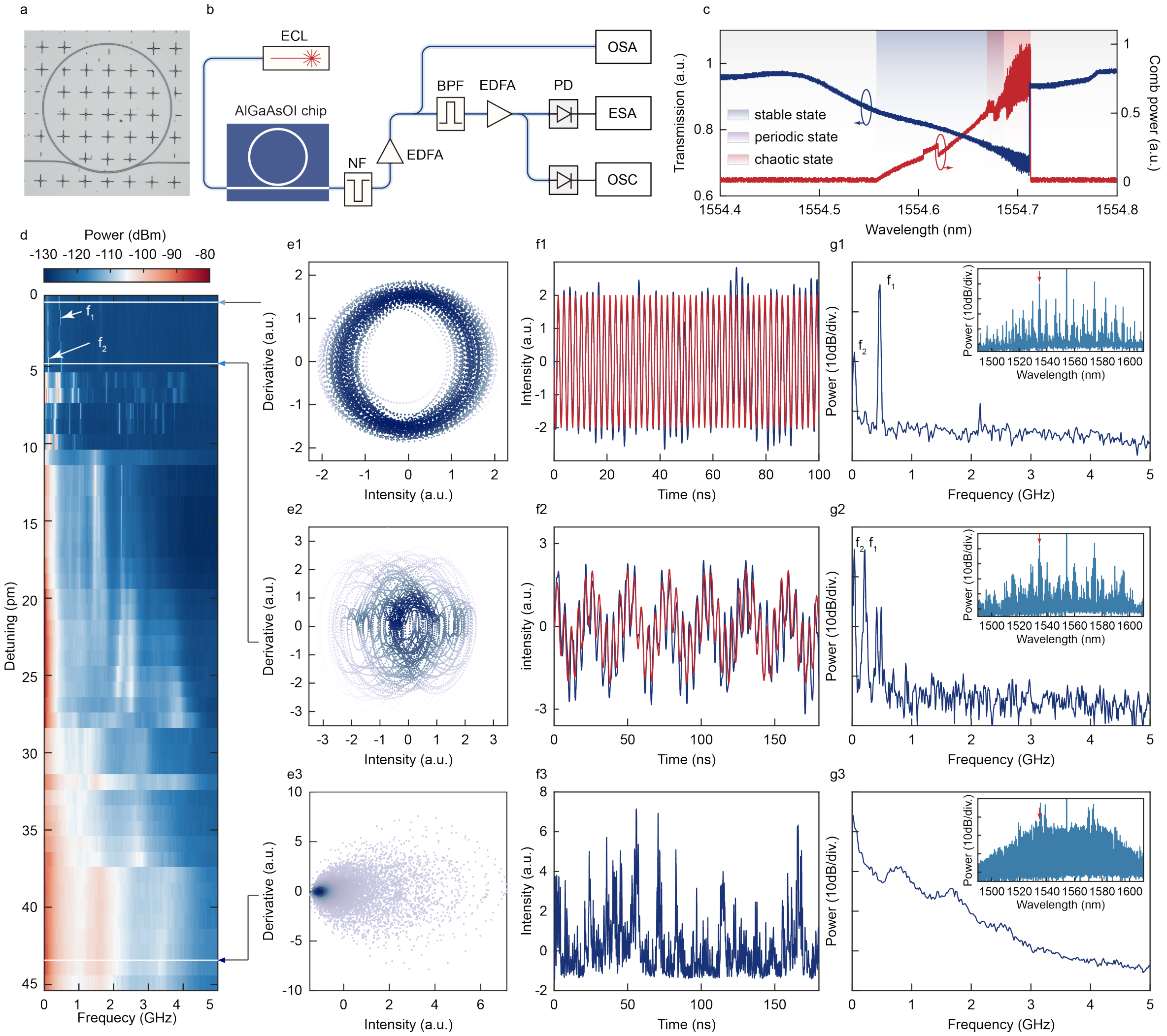

Supplementary Fig. 4: Observation of the route into the chaotic state. a, Optical microscope photograph of the AlGaAsOI microring. **b,** Setup for observing the chaotic comb generation. ECL, external cavity diode laser; EDFA, erbium-doped fiber amplifier; NF, notch filter; BPF, band-pass filter; WSS, wavelength selective switch; PD, photodetector; OSC, oscilloscope; ESA, electrical spectrum analyzer; OSA, optical spectrum analyzer. **c,** Normalized transmitted total power (blue) and comb power (pink). The shade region where rapid oscillation is observed marks the region for chaotic comb state. **d,** the 2D RF spectral map of the comb line around 1535 nm. **e1-3,** Phase portraits for the single period state (1), the multi-period state (2) and the chaotic state (3). **f1-3,** Time domain variations for different states. **g1-3,** RF spectra for different states. The insets show the optical spectra of different states.

Supplementary note IV Verification of the inter-channel orthogonality

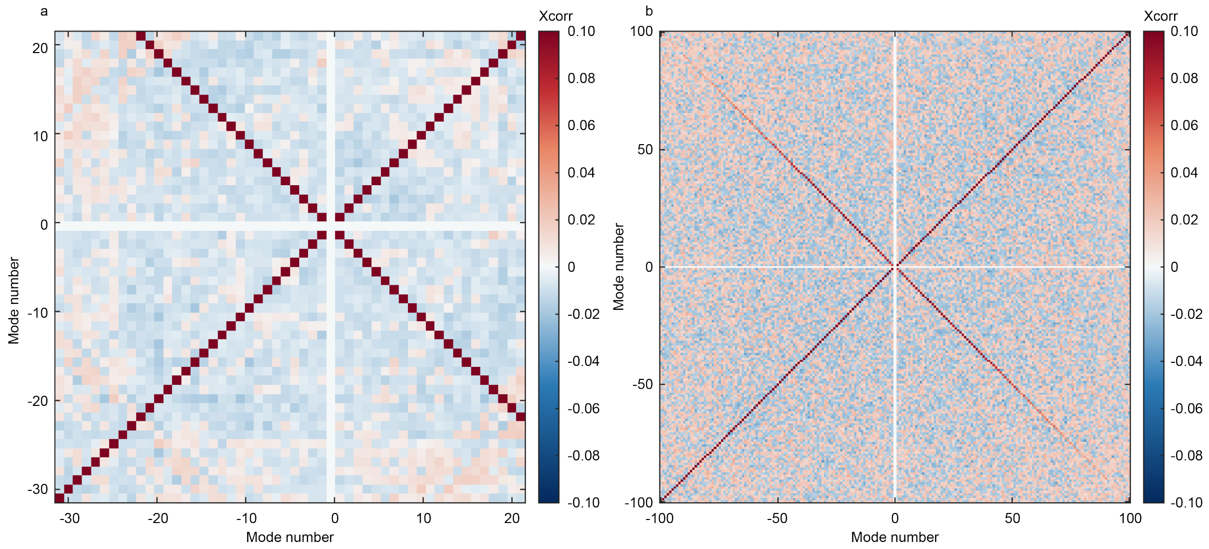

Supplementary Fig. 5: Inter-channel correlation in experiment (a) and in simulation (b).

Shown in Fig. 3h and Supplementary Fig. 5a, the inter-channel correlation is obtained in experiment. It is worth noting that there is an obvious correlation between symmetrical comb lines while the correlation between other comb lines is weak (less than 0.04) enough to be neglected. These properties are also captured in simulation as shown in Supplementary Fig. 5b, where a pump power of around 100 mW is employed.

Supplementary note V Simulation: Influence of different parameters

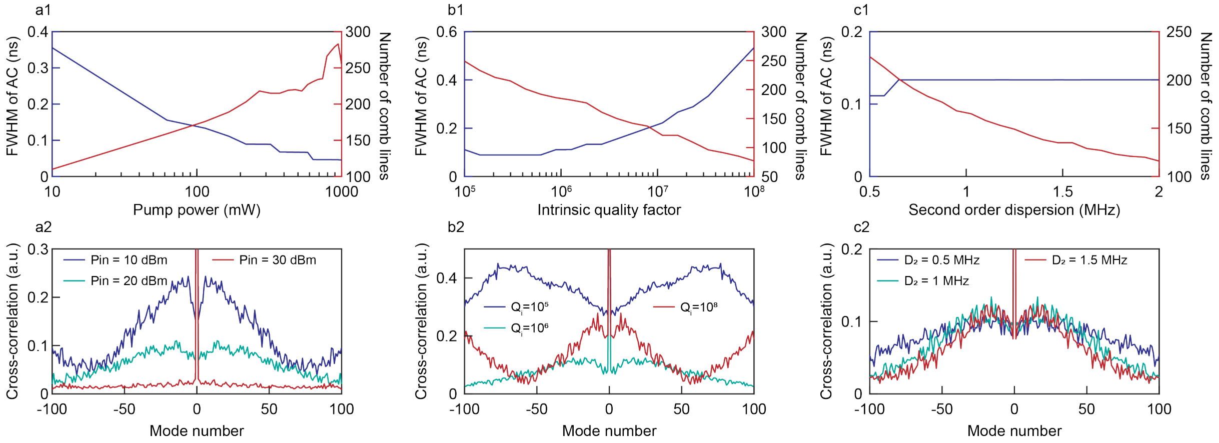

Supplementary Fig. 6: Influence of different parameters on chaotic combs. a1, b1, c1, Full widths at half maximum of the autocorrelation and numbers of comb lines under different pump powers, intrinsic quality factors and second order dispersion. **a2, b2, c2,** the cross-correlation between symmetric comb lines under different pump powers, intrinsic quality factors and second order dispersion.

In this section, we will show the influence of different parameters on the chaotic comb. For simplification, different chaotic combs are characterized by the minimum full widths at half maximum (FWHM) of the autocorrelation of comb lines around the pump mode. Supplementary Fig. 6 shows the results under different conditions. It is worth noting that the FWHM of autocorrelation function would be significantly affected by the quality factor and the pump power, while no obvious variation could be found as the variation of the second order dispersion. By increasing the pump power, the intracavity energy could be rapidly raised, resulting in a more chaotic state. Thus, it is reasonable that the second order dispersion will not influence the FWHM of the ACF. While the number of comb lines will be affected by the second order dispersion as the circumstance of soliton microcombs. Different from the soliton microcomb, as shown in Supplementary Fig. 6b1, it seems that a high-quality factor is not a positive condition for chaotic combs. This can be explained by the effective coupling state as illustrated in [5]. Under a low pump power where the optical nonlinear effect is not strong enough, the highest intracavity power is obtained under a critical coupling state, where the coupling factor is the same as the loss factor. It is not the same under a high pump power where the energy conversion from the pump mode to other

modes is induced by the optical nonlinearity. The nonlinear conversion will induce effective nonlinear loss on the pump mode, resulting in an effective under-coupling state, decreasing the intracavity field. Thus, a proper over-coupling state will be more suitable to obtain a better chaotic comb.

Supplementary note VI Simulation: Influence of coupling state

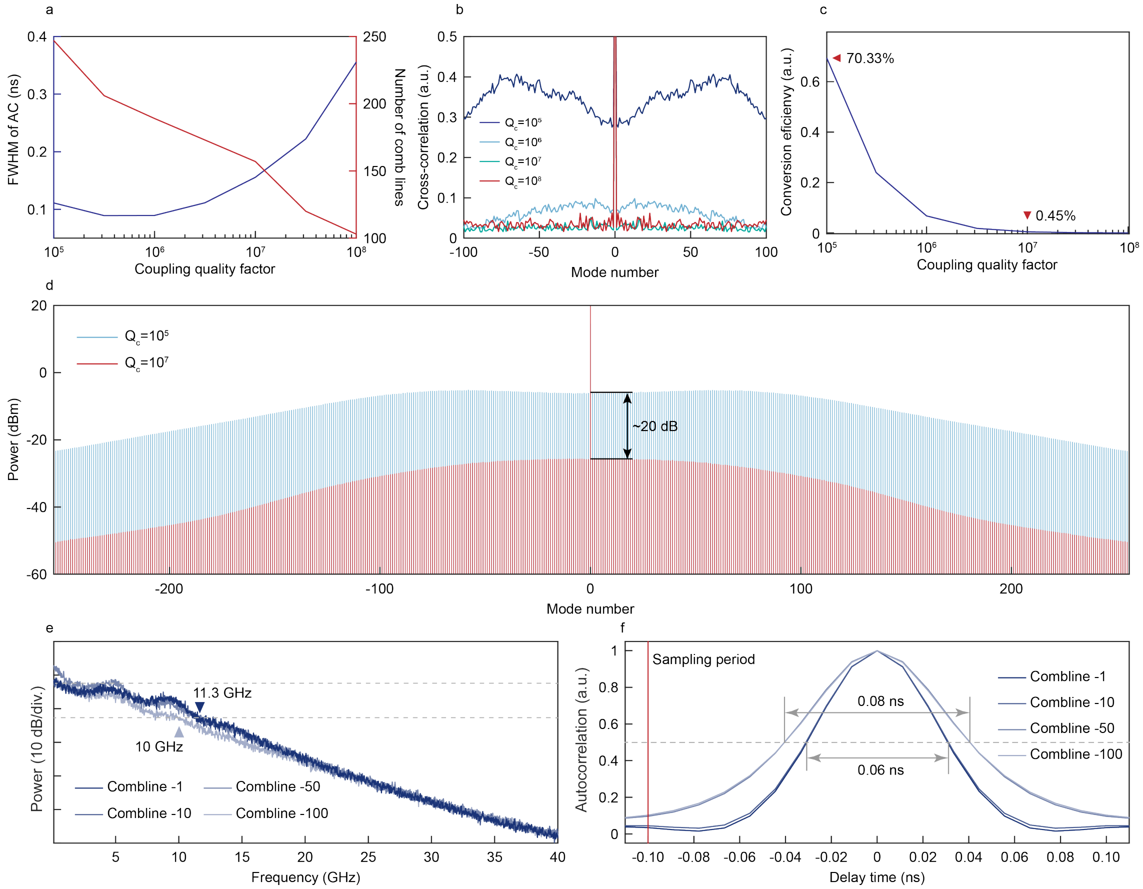

Supplementary Fig. 7: Influence of the coupling state on chaotic combs. a, the FWHM and the number of comb lines under different coupling states. **b,** the XCF between symmetric comb lines around the pump mode. **c,** the conversion efficiency under different coupling states. **d,** the optical spectra under over-coupling state ($Q_c = 10^5$) and critical-coupling state ($Q_c = 10^7$). RF spectra (**e**) and ACFs (**f**) of different comb lines under the over-coupling state.

Supplementary Fig. 7 shows properties of chaotic combs under different coupling states, where the coupling quality factors are varied from 10^5 to 10^8 with the intrinsic quality factor set to 10^7. Under an over-coupling state ($Q_c < 10^7$), a lower FWHM of ACF is ensured compared with the critical coupling state, indicating a better chaotic state. A higher conversion efficiency is also attractive as a higher comb line power is promised. Compared with the critical coupling state, the over-coupling state can deliver comb lines with 20 dB higher power. Supplementary Fig. 7e and f show the radio frequency spectra and

autocorrelation functions of different comb lines (with mode number -1, -10, -50, -100 as shown in Supplementary Fig. 7d). It is worth noting that all comb lines feature an effective radio-frequency bandwidth above 10 GHz and an FWHM below 0.1 ns. Considering a sampling rate of 10 GSa/s, the correlation between adjacent sampling data is around 0.1, which is acceptable for a random bit generator. With a proper design, the total output rate of the chaotic-comb-based parallel random bit generator could reach 3 Tbps (considering 30 Gbps pre-channel).

Supplementary note VII Experiment and Simulation: Influence of the inter-mode coupling

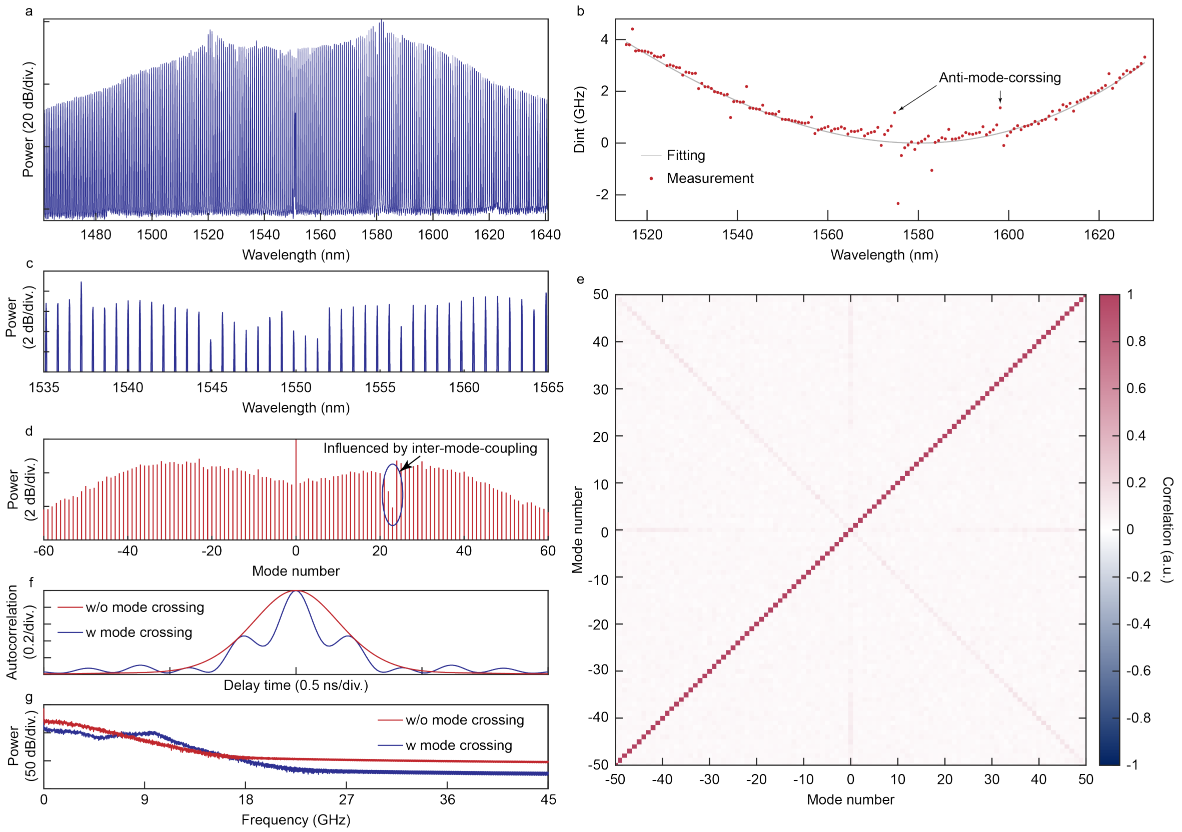

Supplementary Fig. 8: Influence of inter-mode coupling. a, the optical spectrum of chaotic comb in the experiment shown in the main text. **b,** the integrated dispersion profile of the tested microring. **c,** the zoom-in optical spectrum at C band. **d,** the simulated optical spectrum considering the mode-crossing. **e,** the correlation between different comb lines in simulation. The auto-correlation function (**f**) and radio frequency spectrum (**g**) with (blue) and without (red) mode crossing.

In Supplementary Fig. 7d, we can see flat optical spectra of chaotic combs, while the optical spectra observed in the experiment were fluctuating at the central part. Supplementary Fig. 8c shows the zoom-in spectrum, where the power variation is approximately 7 dB. The power variation could be induced by inter-mode coupling as the circumstance in the bright soliton. Supplementary Fig. 8b shows the measured integrated dispersion profile of the tested microring, where multiple anti-mode-corssing points can be observed. In the simulation, more than one transverse mode can be supported in the waveguide used here

(400 nm $\times$ 650 nm). Coupling between different modes is possible as orthogonality between modes is not promised, causing shifted resonance frequency and varied quality factors. The influence of inter-mode coupling has been well studied in [6, 7]. Summarily, the inter-mode coupling can induce power variation at comb lines of the chaotic comb. Supplementary Fig. 8d shows a simulated optical spectrum where an anti-mode-crossing is induced around mode 23. Supplementary Fig. 8e shows the correlation between different comb lines considering the inter-mode coupling. Despite localized dispersion variation, the great orthogonality between comb lines is not lost, agreeing with our experiment result. The autocorrelation function and radio-frequency spectrum at the crossing point are given in Supplementary Fig. 8f and Supplementary Fig. 8g. In this simulation case, it is worth noting that the presence of mode crossing results in a wider RF spectrum. This wider spectrum may be attributed to the broader resonance and lower quality of the microcomb caused by the mode crossing. The impact of mode crossing on microcombs is a complex problem, which requires further investigation in future work.

Supplementary note VIII Simulation: Influence of three-photo-absorption

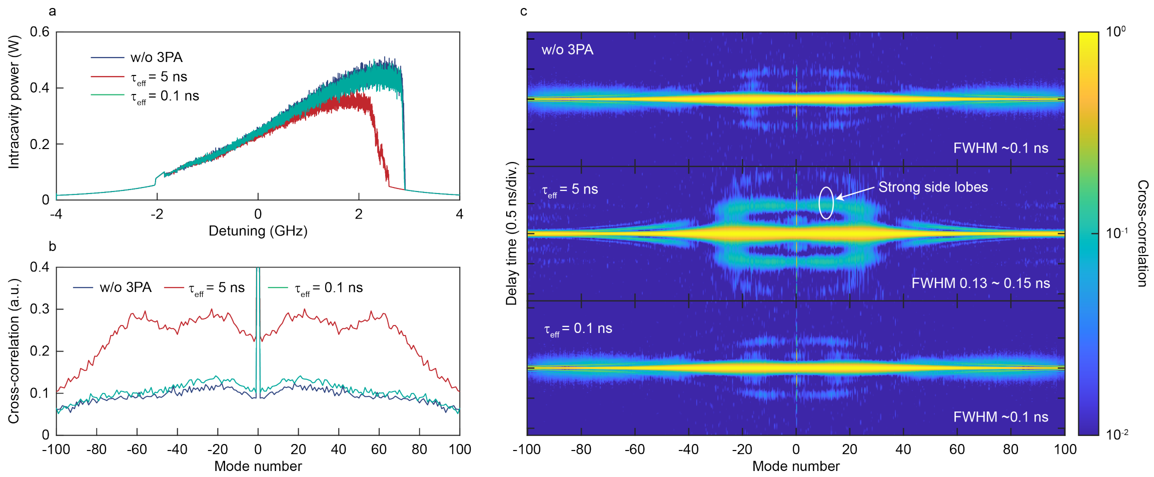

Supplementary Fig. 9: Influence of the three-photon-absorption and the free carrier absorption. **a,** the total intracavity power evolution with the detuning under different conditions of the 3PA. The blue line represents the microcomb evolution without 3PA. The red and green lines are the evolution with 3PA and FCA where the effective carrier lifetime is 5 ns and 0.1 ns respectively. **b,** the cross-correlation between symmetric comb lines. **c,** the autocorrelation function of comb lines around the pump mode. The top, middle and bottom planes are the ACF map without 3PA, with 3PA while the effective carrier lifetime is 5 ns and 0.1 ns respectively.

In this work, a high-quality chaotic state is promised by the remarkable nonlinear coefficient of the AlGaAsOI platform and a high pump power up to 130 mW. Getting rid of the TPA, the component of the AlGaAs is set to $Al_{0.2}Ga_{0.8}As$. However, under a pump power up to hundreds of microwatts, the influence of the three-photon-absorption (3PA) and the corresponding free-carrier-absorption (FCA) should be considered. Supplementary Fig. 9 shows the influence of the 3PA and the FCA on the chaotic properties. To take the 3PA into account, a modified Lugiato-Lefever equation with the equation describing the dynamics of the free carrier is employed:

$$t_R\frac{\partial E}{\partial t} = \left[-\left(\frac{\alpha}{2}-i\delta_0\right)+iL\frac{\beta_2}{2}\frac{\partial^2}{\partial\tau^2}\right]E-\left[\frac{\beta_{3PA}L}{3A_{\text{eff}}^2}|E|^4+\frac{\sigma L}{2}(1+i\mu)N_c\right]E+iL\gamma|E|^2E+\sqrt{\theta}E_{in}$$
$$\frac{\partial N_c}{\partial\tau} = \frac{\beta_{3PA}}{3\hbar\varpi}\frac{|E|^6}{A_{\text{eff}}^3}-\frac{N_c}{\tau_{\text{eff}}} \quad (5)$$

where the 3PA and free carrier absorption are considered as the second part at the right side of the Eq. S4. β_{3PA} is the 3PA coefficient, A_{eff} is the effective area of the waveguide, σ and μ are the FCA parameters, N_c is the density of free carriers and τ_{eff} represents the effective life of free carriers. Considering an effective carrier lifetime of 5 ns, the chaotic signal will be degraded. As for the cross-correlation between symmetric comb lines, the correlation raised compared with the chaotic comb without the 3PA. More seriously, the autocorrelation property is also degraded with a larger FWHM and higher sidelobes. All this degradation can be attributed to a lower intracavity power as shown in Supplementary Fig. 9a. The simulation result can explain the difference between our experiment result where the FWHM under 130 mW is higher than that in simulation under the same condition. This indicates the chaotic property of chaotic combs in AlGaAsOI microrings is limited by the 3PA. This could be eased by inducing the PIN junction structure to shorten the effective carrier lifetime, which has been well studied in nonlinear optics based on Silicon-on-Insulator platform [8]. Considering an effective carrier lifetime of 0.1 ns, the chaotic signal is of the same quality as that without 3PA. The AlGaAsOI microring with PIN junction structures might be a more excellent platform for the chaotic comb generation, to deliver massively parallel chaotic signals with tens GHz chaotic bandwidth.

Supplementary note IX Experiment: Details of the test link

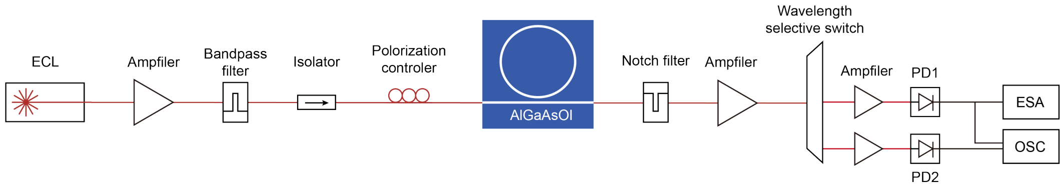

Supplementary Fig. 10: The test link of chaotic combs

Supplementary Fig. 10 shows the general test link for the experiment. To stimulate the chaotic comb state, a high quality AlGaAsOI microring is pumped. To get a high pump power, an EDFA is employed to boost the pump laser to a high level and a bandpass filter is used to suppress the ASE noise. Then the amplified pump laser is injected into the microring after passing an isolator and a polarization controller. As illustrated above, by sweeping the frequency of the pump laser, we could obtain a chaotic comb as a massively parallel chaotic source. At the output side of the microring chip, a notch filter is used to suppress the pump mode and the remaining comb lines are boosted by a DWDM EDFA. Then the amplified comb lines are divided by the wavelength selective switch and sent to different photodetectors . Due to the insertion loss induced by the wavelength selective switch (6 dB), an EDFA is used before injecting into the PD. The detected signal is sent into an oscilloscope to observe the time domain variation and an electrical spectrum analyzer to obtain the RF domain spectrum. For the characterization of the cross-correlation, two comb lines are filtered and sent to two PDs respectively at the same time. Cross-correlation is employed to the two detected signals to value the correlation between two selected channels.

In the application for random bit generation, a silicon photonic wavelength division demultiplex receiver is employed. Fig. 3 shows the test link and Supplementary Fig. 11a shows the microscope image of the SiPh chip. The DWDM receiver is constituted of one 16-channel arrayed waveguide grating and 16 Ge-on-Si photodetectors. The optical loss from the input edge coupler to the photodetector is estimated to be 8∼9 dB, by testing a similar circuit where photodetectors are replaced by an array of edge couplers. As the amplified comb line is injected into the chip, the AWG will selectively divide the input signal into PD. The tested transmission spectra of the 16-channel AWG are given in Supplementary Fig. 11b. The channel spacing is verified to be around 180 GHz, fit well with the channel spacing of the microcombs.

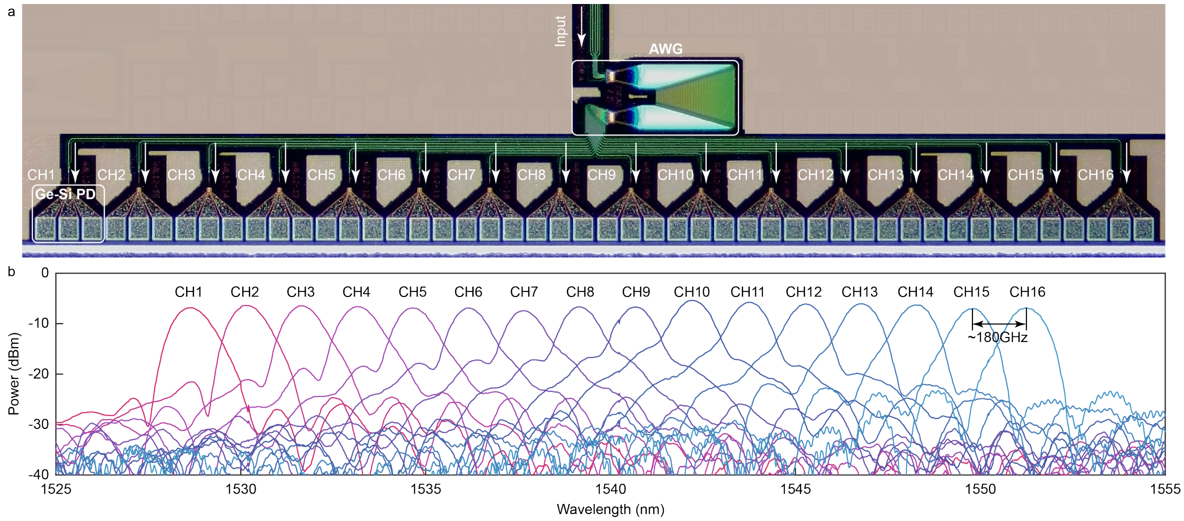

Supplementary Fig. 11: SiPh wavelength division demultiplex receiver. a, the microscope image of the SiPh chip. **b,** transmission spectra of the 16-channel 180 GHz AWG.

Supplementary note X Experiment: Comparison of commercial InP photodetectors and Si-Ge photodetectors

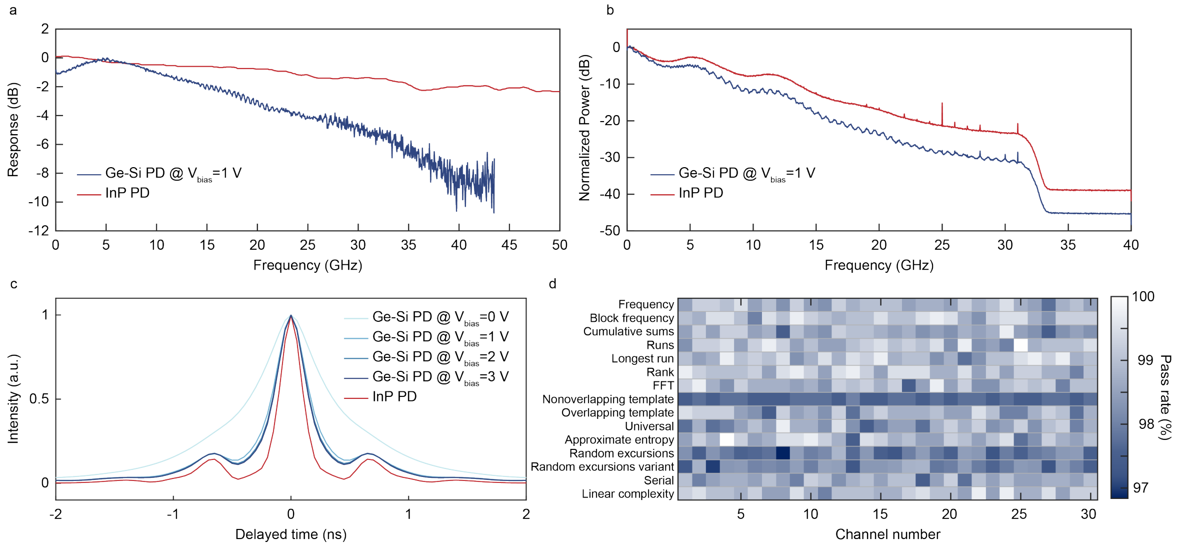

Supplementary Fig. 12: Comparation of integrated Ge-Si photodiode and commercial InP photodiode. a, the measured frequency response of the Ge-Si photodiode and InP photodiode. **b,** the frequency spectra of signals detected by Ge-Si photodiode and InP photodiode. **c,** Autocorrelation functions of chaotic signals detected by Ge-Si photodetectors and commercial InP photodetectors. **d,** the NIST SP 800-22 test results for random bits generated by InP photodiode with 30 Gbps generation rate.

In the experiment, the chaotic signal is detected by commercial InP photodetectors and Si-Ge photodetectors respectively. In this part, we show that the frequency response or bandwidth of the Ge-Si photodiode is worse than that of the commercial photodiode, which degenerates the recorded signal. Supplementary Fig. 12a shows the measured frequency response of the integrated Ge-Si photodiode and the InP photodiode. The Ge-Si photodiode shows a faster decrease at the high frequency. This is revealed at the frequency spectra of recorded signal as shown in Supplementary Fig. 12b. Supplementary Fig. 12c shows the autocorrelation function of the signal of the same comb line detected by the Ge-Si photodetector and the commercial InP photodetector employed in the main part. At different reverse biases, the detected signals express different features due to the relationship between the bias voltage and the bandwidth. The FWHM detected by the InP photodetector is 0.22 ns. While the FWHM detected by the Ge-Si photodetector is about 0.30 ns, due to the inferior frequency response. Employing the data recorded by the InP photodiode, the generation rate could

reach 30 Gbps and the generated random bit sequences can pass the NIST SP 800-22 test successfully. In addition, it is worth noting that the input power of -9 dBm is enough for the Ge-Si photodetector as illustrated in [9]. Considering a high conversion efficiency shown in Supplementary Fig. 6d where the power of each comb line could reach -5 dBm, a fully integrated chaotic signal detector without an inter-chip amplifier is possible.

Supplementary note XI Diehard test result for random bit generation

Test	1	2	3	4	5	6	7	8	9	10	11	12	13	14	15
Birthday spacing (KS)	0.6885	0.9983	0.8438	0.5353	0.9171	0.8438	0.5385	0.5601	0.8285	0.3538	0.5413	0.1938	0.7019	0.1991	0.3641
Overlapping 5-permutation	0.1465	0.7644	0.5995	0.0025	0.0483	0.1672	0.8468	0.0042	0.7084	0.3354	0.7482	0.0111	0.1026	0.3284	0.2853
Binary rank (32 x 32)	0.5586	0.7462	0.9722	0.6419	0.8486	0.1521	0.8130	0.7204	0.2659	0.7093	0.8851	0.0342	0.7266	0.3052	0.0285
Binary rank (6 x 8) (KS)	0.8350	0.6990	0.6863	0.9088	0.4228	0.3185	0.4963	0.2617	0.4782	0.9461	0.9535	0.2619	0.1798	0.7352	0.8734
Bitstream	0.7664	0.0446	0.3066	0.2655	0.4425	0.6597	0.1623	0.9163	0.8966	0.9369	0.4674	0.3149	0.2943	0.8892	0.1408
OPSO	0.0036	0.6025	0.0698	0.1709	0.0065	0.8130	0.0747	0.0817	0.0109	0.6225	0.0063	0.0095	0.3413	0.5056	0.0768
OQSO	0.0030	0.1564	0.0775	0.7432	0.9739	0.1626	0.7112	0.8274	0.0763	0.1764	0.0481	0.2173	0.7711	0.0415	0.6673
DNA	0.0412	0.7616	0.7931	0.5394	0.0683	0.0770	0.3617	0.9119	0.8321	0.3947	0.4402	0.6584	0.9142	0.3716	0.9867
Count-the-1's, a stream of bytes	0.3101	0.5641	0.4102	0.5305	0.5188	0.1348	0.0992	0.9424	0.9763	0.6254	0.3195	0.4739	0.2144	0.9785	0.3318
Count-the-1's, specific bytes	0.4306	0.7696	0.8981	0.2982	0.7049	0.1642	0.7995	0.3168	0.7291	0.2723	0.8973	0.6272	0.6009	0.9645	0.6023
Parking lot (KS)	0.9474	0.4360	0.6967	0.9845	0.8822	0.3462	0.1149	0.8871	0.6306	0.7960	0.5952	0.1462	0.4300	0.1530	0.4300
2D-spheres (KS)	0.6127	0.0622	0.4113	0.9319	0.3028	0.5786	0.6290	0.5573	0.6941	0.2034	0.3413	0.9779	0.9167	0.7527	0.2283
3D-spheres (KS)	0.5783	0.7118	0.0515	0.5133	0.2838	0.9227	0.8697	0.5292	0.3329	0.0469	0.1975	0.9168	0.9987	0.4261	0.8636
Squeeze	0.2448	0.2318	0.0581	0.0287	0.0445	0.0034	0.0146	0.0080	0.4683	0.0092	0.4514	0.1107	0.2934	0.3180	0.3166
Overlapping sums (KS)	0.0264	0.3108	0.0175	0.0160	0.2468	0.3056	0.0139	0.4497	0.2509	0.0007	0.1179	0.6970	0.0425	0.0358	0.0220
Runs (KS)	0.0899	0.2488	0.0683	0.0101	0.8129	0.0786	0.3939	0.0170	0.6432	0.3586	0.2898	0.5924	0.0262	0.3799	0.6784
Craps	0.0898	0.7718	0.9047	0.9916	0.6134	0.9814	0.7556	0.8407	0.6396	0.8917	0.9400	0.9671	0.7623	0.6166	0.2067

Channel number

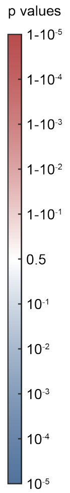

Supplementary Fig. 13: Autocorrelation functions of chaotic signals detected by Ge-Si photodetectors and commercial InP photodetectors.

To evaluate the random bit generators based on chaotic combs, the Diehard tests are employed here, except for the NIST tests used in the main part. For each test, one or multi p-values will be given, to access the quality of random bits. A p-value close to 0 or 1 indicates bad quality. Considering a 95% confidence interval, p-values between 10^{-4} and $1-10^{-4}$ mark the random bit generator could pass the Diehard test. The test results of random bits extracted using Ge-Si photodetectors are shown in Supplementary Fig. 12. All 15 channels could pass the test, showing reliable randomness.

Supplementary note XII The influence of different amplification processes

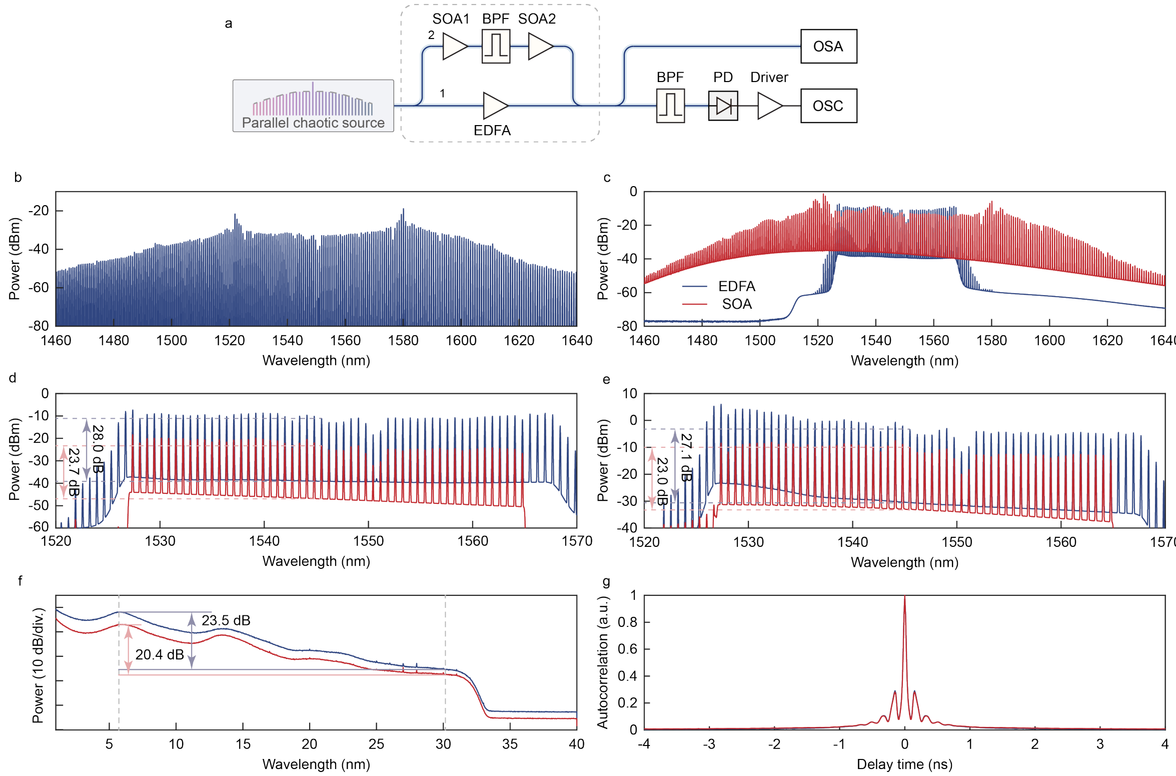

Supplementary Fig. 14: The influence of different amplification processes. a, the schematic of the test link. **b,** the optical spectrum before amplification. **c,** the optical spectra after the first order amplification. **d,** the optical spectrum after the first order amplification around the C-band. The spectrum of SOA is filtered by a band pass filter. **e,** The optical spectrum after the second order amplification. **f,** the radio-frequency spectra of the recorded comb line. **g,** the autocorrelation functions of the recorded comb line.

In the experiment described in the main text, we used a commercial EDFA to amplify the generated chaotic comb due to its weak comb line power. Here, we compare the amplification performance of commercial EDFAs and semiconductor optical amplifiers (SOAs). Supplementary Fig. 14 shows the experimental setup. Unlike the main text, where an optical amplifier was arranged between the bandpass filter and the photodiode, an electrical amplifier is arranged after the photodiode to amplify the detected signal and compensate for the limited response of the commercial InP photodiode. First, we record the original optical spectrum of the chaotic comb, as shown in Supplementary Fig. 14b. The signal-to-noise ratio (SNR) of comb lines within the C-band can reach 40 dB. Supplementary Fig.

14c shows the spectra of amplified combs. The first order amplification of the EDFA is set to the maximum. Different from the EDFA, the gain bandwidth of the SOA is wider. To obtain higher power in the C-band, a bandpass filter was employed after the first SOA to suppress comb lines outside the C-band. The filtered spectrum is shown in Supplementary Fig. 14d. Due to a higher noise figure compared to the EDFA, the comb lines amplified by the SOA show lower SNR. For the comb line around 1545 nm, the SNR is 23.7 dB, which is 4.3 dB lower than that amplified by the EDFA. Moreover, due to the insertion loss of the bandpass filter (5 dB), the power amplified by the SOA is lower. Thus, the filtered comb is amplified by another SOA. Supplementary Fig. 14e shows the optical spectrum after the second SOA. In addition, for the EDFA, the second-order amplifier is turned on and set to the maximum. The output powers of the SOA and the EDFA are 16 dBm and 22 dBm, respectively. Thus, the comb line amplified by the SOA is weaker. One comb line around 1545 nm is filtered out by a tunable bandpass filter with 6 dB insertion loss. The comb line power sent to the photodiode is -3 dBm and -8 dBm for the EDFA and SOA, respectively. Supplementary Fig. 14f shows the radio frequency spectra amplified by the EDFA and SOA. Due to a higher noise figure and lower saturation power, the signal amplified by the SOA shows a 3.1 dB lower SNR. Supplementary Fig. 14g shows the auto-correlation function of the comb line amplified by the SOA and EDFA respectively. The two lines are almost coincident with each other. Thus, at the present set up, the random from the chaotic comb is not corrupted by the replacement of EDFA with SOA.

Supplementary note XIII Parameter set of decision makers for multi-armed bandit problem

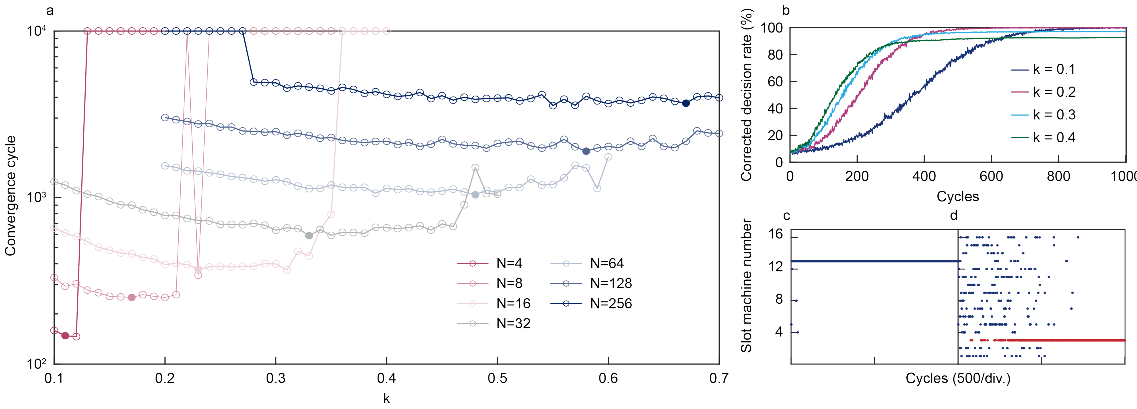

Supplementary Fig. 15: The selection of k. a, the convergence cycle under different values of k for different problem scales. Solid dots are k values employed for decision makers in the main part. **b,** the correct decision rate evolution with cycles under different k values, with N=16. **c (d)**, one decision process under k=0.4 (0.1).

As we have discussed in Methods, the selection of k value is vital for decision makers. Thus, we sweep the k value for different problem scales, and the convergence cycles are recorded as shown in Supplementary Fig. 15a. To show the influence of k on the convergence speed, the evolution of the corrected decision rate with the increase of cycles under different k values are shown in Supplementary Fig. 15b, where N=16. It is obvious that a higher convergence speed is accompanied with a larger k at the beginning of the decision process. While the corrected decision rate could not reach 100% as the game carrying on. It is caused by worry decisions as shown in Supplementary Fig. 15c, where one decision process with k=0.4 is presented. Due to a large k, the decision maker only tests 5 slot machines and a high bias value is added to the 25th channel, making a premature decision without sufficient exploration. In addition, one decision process with k=0.1 is shown in Supplementary Fig. 15d. Under a small k, the decision maker will be more cautious, causing a slow convergence speed. Thus, as for different scale, we adopt the k value where the convergence cycle is the smallest. K values employed in the main part are marked with solid dots as shown in Fig. S12a, where k=0.11, 0.17, 0.23, 0.33, 0.48, 0.58, 0.67 respective for N=4, 8, 16, 32, 64, 128, 256.

Supplementary References

[1] Hart, J. D. *et al.* Recommendations and illustrations for the evaluation of photonic random number generators. *APL Photonics* **2**, 090901 (2017).

[2] Lugiato, L. A. & Lefever, R. Spatial dissipative structures in passive optical systems. *Physical review letters* **58**, 2209 (1987).

[3] Panajotov, K., Clerc, M. G. & Tlidi, M. Spatiotemporal chaos and two-dimensional dissipative rogue waves in lugiato-lefever model. *The European Physical Journal D* **71**, 1–8 (2017).

[4] Leo, F., Gelens, L., Emplit, P., Haelterman, M. & Coen, S. Dynamics of one-dimensional kerr cavity solitons. *Optics express* **21**, 9180–9191 (2013).

[5] Xue, X., Wang, P.-H., Xuan, Y., Qi, M. & Weiner, A. M. Microresonator kerr frequency combs with high conversion efficiency. *Laser & Photonics Reviews* **11**, 1600276 (2017).

[6] Guo, H. *et al.* Intermode breather solitons in optical microresonators. *Physical Review X* **7**, 041055 (2017).

[7] Yi, X. *et al.* Single-mode dispersive waves and soliton microcomb dynamics. *Nature communications* **8**, 1–9 (2017).

[8] Yu, M., Okawachi, Y., Griffith, A. G., Lipson, M. & Gaeta, A. L. Mode-locked mid-infrared frequency combs in a silicon microresonator. *Optica* **3**, 854–860 (2016).

[9] Liu, Z. *et al.* 25$\times$ 50 gbps wavelength division multiplexing silicon photonics receiver chip based on a silicon nanowire-arrayed waveguide grating. *Photonics Research* **7**, 659–663 (2019).
